# A feasibility study on predicting cow calving time over 40 h in advance using heart rate and financial technical indicators

**DOI:** 10.1038/s41598-024-72521-w

**Published:** 2024-09-18

**Authors:** Shigeki Kishi, Tomoki Kojima, Chen-Yu Huang, Ken-ichi Yayou, Kouki Fujioka

**Affiliations:** 1 Research Center for Agricultural Information Technology, National Agriculture and Food Research Organization, 2-14-1 Kowa-Nishi-Shimbashi Building, Nishi-Shimbashi, Minato, Tokyo, 105-0003 Japan; 2 Institute of Livestock and Grassland Science, National Agriculture and Food Research Organization, 2, Ikenodai, Tsukuba, Ibaraki 305-0901 Japan

**Keywords:** Calving, Heart rate, Financial technical indicator, Simple moving average, Biological techniques, Cardiology

## Abstract

In dairy farming, the uncertainty of cow calving date often imposes waiting costs for days on farmers. Improving the accuracy of calving date prediction would mitigate these costs, specifically before a few days of the event. We monitored and analyzed the heart rate patterns of eight pregnant cows in the days leading up to calving using a dedicated monitoring device. We decomposed the heart rate data into three distinct components: trend, daily cycle, and the remainder, and discovered that the heart rate trend exhibited a sharp decline more than 40 h before the calving event via the trend turning point. To detect the turning point, we applied common financial technical indicators traditionally used to identify turning points of asset prices in trading markets for the extracted heart rate trend. This study remains a feasibility study because of the limited observations, but it indicates that these indicators can effectively capture the trend’s turning point in real time, offering a promising approach for enhanced calving prediction. In addition to discussing the practical implications for cow management, we also contemplate the broader utility of these technical indicators in the context of various dynamic scientific data analyses.

## Introduction

Parturition imposes significant stress and costs on the cow, the calf, and the farmers^1^. This event often results in adverse effects on both the calf and mother^2^. While calving dates are typically predicted based on a gestation length of approximately 280 days after breeding, the actual event frequently deviates by a few days due to genetic and physiological factors^3^. Consequently, many cattle farmers experience heightened anxiety in the days leading up to calving as they prepare to assist the mother during this critical period^4^. Although farmers may empirically recognize impending calving by observing certain external indicators of a mother cow^5^, achieving more precise predictions during the last week could significantly reduce the associated risks and financial and labor costs and enhance overall herd management.

Several studies have explored Internet of Things (IoT) devices and video recorders to detect physiological and behavioral changes just before calving as a predictive signal^4,6,7^. For example, a decline in vaginal temperature during the final stages of pregnancy^8^, and behavioral changes, such as reduced rumination duration^9^ and increased lying time, head turns, and stamping during the final 2 hours^10^, have been reported. However, these changes usually occur within 24 h before the calving and do not always contribute to more accurate predictions^11^. Therefore, there is a need for an earlier and simpler alerting system.

In this study, we focused on monitoring the heart rate (HR) of pregnant individuals. HR and the heart rate variability (HRV), along with related indices such as low-frequency (LF) and high-frequency (HF) components, as well as the LF/HF ratio, have been recognized as indicators of stress, pain, and energy expenditure in livestock^12,13^. These metrics are associated with behavioral patterns and sympathetic and vagal activities^12,13^. A study that monitored HR and HRV during successive calving stages identified an increase in HR between 12 and 24 h before calving^14^. If this HR increase is a sign of calving, it could enable us to predict the precise delivery date and time.

Monitoring HR using simple devices and the recorded data is more accessible for farmers than video-monitoring systems and their data^15^. Using a Holter monitor device (QR2500; Fukuda ME Co. Ltd, Tokyo, Japan), we recorded R-R intervals, which are periods between two adjacent peaks of R wave and can be converted to HR using a simple formula, HR (bpm) = 60/R-R interval (seconds), of eight individual cows for about one week leading up to the expected calving day. However, data from two pregnant individuals were excluded from the main analyses due to partial data loss in device troubles (h019 and h873, see Supplementary Information).

At first, we decomposed the recorded R-R intervals into trend, seasonality, and remainder by using a seasonal and trend decomposition procedure based on LOESS (locally estimated scatterplot smoothing curve) (STL analysis)^16^. The seasonality component represents a daily cyclic fluctuation in R-R intervals. Using financial technical indicators, we explored using the turning points of the extracted trend as promising indications that calving becomes imminent. Many technical indicators have long been used by financial market traders and investors who must quickly decide whether to buy or sell financial products, such as stocks, bonds, currencies, commodities, and options, while those prices fluctuate^17^. In this study, we used three common indicators: Moving Average Deviation Rate (MAD), Moving Average Convergence/Divergence (MACD)^18^, and Relative Strength Index (RSI)^19^ (Table 1). Our purpose here is not to discuss whether price trends exist in the financial market^20,21^. Rather, we examined whether these indicators could be used to predict change points in heart rate and thus the remaining time to calving.

**Table 1:** Calculation formulas and summary of financial technical indicators.

Indicator	Formula	Summary
Simple moving average (SMA)	${SMA}_{n,t}=\frac{{P}_{t}+{P}_{t-1}+\dots +{P}_{t-n+1}}{n}$	A price of simple moving average SMA at the time, *t*, is the average of the past *n* (= 12) closing prices (*P*_*t*_, …, *P*_*t–n-1*_)
Moving average deviation rate (MAD)	${MAD}_{n,t}={P}_{t}-{SMA}_{n, t}$	The MAD value at the time, *t*, is the price at the time, *t*, minus SMA (*n* = 12) at the time, *t*
Moving average convergence /Divergence (MACD)	${EMA}_{n,t}={P}_{t}\frac{N+1}{2}+{EMA}_{n,t-1}$ ${MACD}_{short, long,t}={EMA}_{short,t}-{EMA}_{long,t}$	The MACD value is the difference between short EMA (*n* = 9) and long EMA (*n* = 26)
Relative strength index (RSI)	${RSI}_{n}=100\frac{{Upward}_{n}}{{{Upward}_{n}+Downward}_{n}}$	The RSI value is the ratio of the upward change to the total upward and downward changes (both absolute values) during the period (*n* = 14)

## Results

**Fig. 1:**
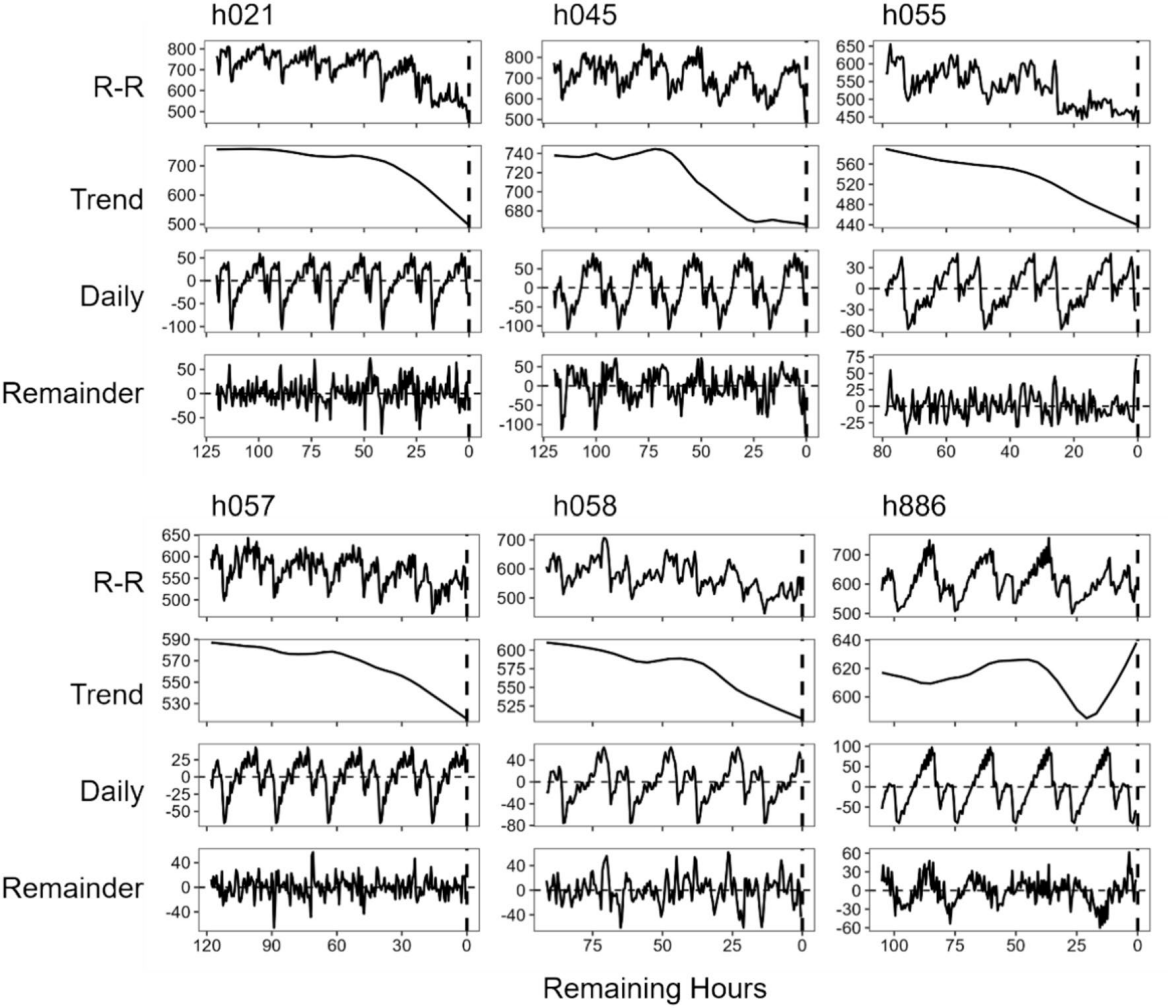
Time series data of R-R intervals (heart-beat intervals, milliseconds) recorded till the calving of every individual (upper row), and its trend (second upper row), daily cycle (second lower row), and the remainder (lower row) decomposed by STL analysis (Seasonal-trend decomposition using LOESS (locally estimated scatterplot smoothing curve)).

STL analysis decomposed the time series data of R-R intervals into the trend, the daily fluctuation, and the reminders (Fig. 1). In all six individuals, the trend line sharply dropped dozens of hours before calving. Before the descent, the trend line exhibited an ascending phase in five individuals, except for h055.

**Fig. 2:**
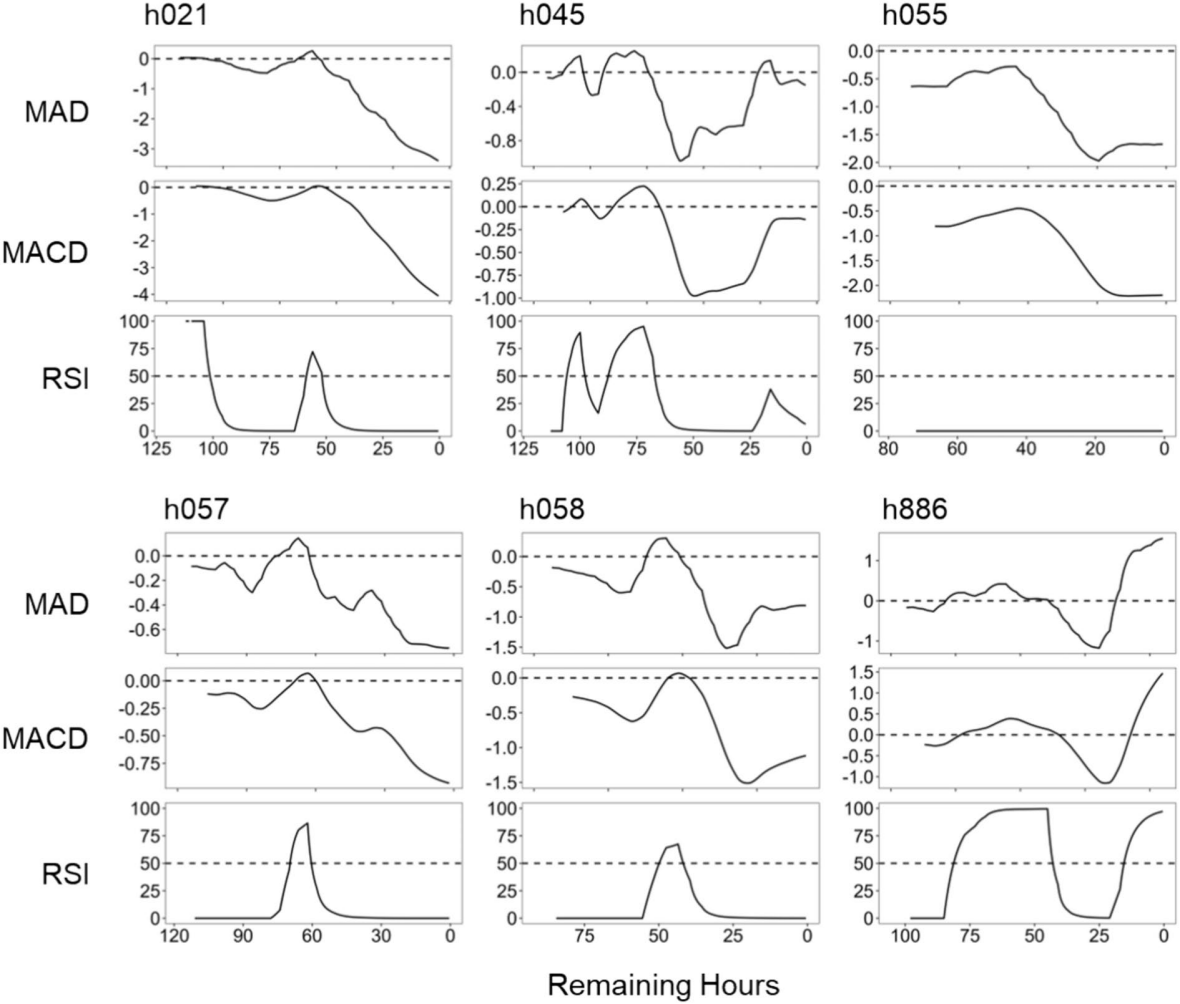
Moving average deviation rate (MAD, the first row), moving average convergence/divergence (MACD, second row), and relative strength index (RSI, third row). The horizontal dashed line was drawn at zero in MAD and MACD, and at 50 in RSI.

All technical indicators detected the turning point in the trend lines extracted by STL analysis except for RSI in h055 (Fig. 2). MAD and MACD exhibited similar patterns, although MACD displayed smoother lines than MAD. Both indicators shifted into positive territory (> 0) before the sharp decline except for h055. Conversely, RSI formed a pointed peak in h021, h057, h058, and h886. In h045, three peaks were observed leading up to calving, while no visible peaks were observed in the RSI in h055. Then MAD, MACD, and RSI indicated the turning point in most cases.

The longest remaining time from the signal points to the onset of calving was 76.0 h for MAD in h045, while the shortest was 42.5 h for MACD in h055 (Table 2). The remaining times for RSI in h055 could not be determined due to the absence of a signal. When omitting missing values, the average remaining time across all three indicators and six individuals was 56.1 ± 11.0 h. In the cases of h021, h045, h055, and h058, the standard deviations of the remaining times, as indicated by the three indicators, were less than 5% of the average values but notably higher at 18.5% in h886.

**Table 2:** Values of financial technical indicators and the remaining hours till calving at the turning point of the R-R (heart-rate) trend.

	h021		h045		h057		h055		h058		h886		
Indicator	Value	Hours	Value	Hours	Value	Hours	Value	Hours	Value	Hours	Value	Hours	Mean ± S.D
SMA12	734.2	53.0	744.2	69.5	578.2	61.0	NA	NA	588.6	42.5	626.1	44.5	54.1 ± 11.3
MAD12	0.3	56.0	0.2	76.0	0.1	66.0	−0.3	43.0	0.3	47.5	0.4	63.5	58.7 ± 12.3
MACD	0.0	53.5	0.2	72.0	0.1	62.0	−0.4	42.5	0.1	43.5	0.4	59.5	55.5 ± 11.4
RSI	72.2	56.0	95.2	72.0	86.5	62.0	NA	NA	67.4	43.5	99.5	45.0	55.7 ± 11.9
Mean ± S.D		54.6 ± 1.6		72.4 ± 2.7		62.8 ± 2.2		42.8 ± 0.35		44.3 ± 2.2		53.1 ± 9.8	

Generalized linear mixed model (GLMM) found a significant difference in the remaining time among the three indicators (*F* = 5.19, *p* = 0.028). Multiple comparison tests with Bonferroni correction detected a significant difference (*p* < 0.0167) between MAD and MACD but no other significant differences (MAD-MACD, *F* = 152.11, *p* < 0.001; MAD-RSI, *F* = 3.64, *p* = 0.13; MACD-RSI, *F* = 0.61, *p* = 0.48). Then, the average remaining time indicated by MAD, 58.7 ± 12.3 (mean ± S.D.), was 3.17 ± 1.44 h longer than that by MACD, 55.5 ± 11.4 (Table 2).

## Discussion

Our study observed a sharp decline in the R-R interval trend, indicating an increase in HR, more than 40 h before calving. We effectively clarified this trend using STL analysis, which helped us extract the trend from the noisy data. We consistently observed convex curves before the sharp decline in all R-R trends, with their peaks as crucial turning points. These turning points can be used as signs, notifying farmers of the impending calving within approximately 50 h. Importantly, this sign is one of the earliest indicators reported in the literature, as previous signs were typically observed within 24 h, such as vaginal temperature and rumination time^4,11^. The signal we identified can give farmers more lead time to prepare for calving. The date predicted by the signal (about two days later) would be more accurate than the date predicted by the breeding date. Then, our method can be used as another prediction tool for the calving date located in the middle between the distant and the proximate prediction tools. Additionally, our method is simple, straightforward, and real-time, ensuring easy use for farmers.

Our investigation into financial technical indicators highlighted their effectiveness in identifying the turning point of the R-R trend. In MAD, based on a simple moving average (SMA), the turning point manifested as a prominent peak above zero (> 0). This peak occurs when the trend line ascends above SMA, resulting in positive values, and rapidly descends below SMA. Even when the peak is less distinct, as observed in the case of h055, the swift descent provides a secondary valuable signal indicating imminent calving. GLMM fitting analysis demonstrated that MAD provided faster notification of the turning point compared to MACD. This result should be attributed to the inherent time delay in MACD because the MACD value is a differential between two exponential moving averages (EMAs) fluctuating behind the original value. In contrast, MAD is a direct differential between the observed value and a simple moving average, then concurrently fluctuating with the observed value. While effectively expressing the peak, RSI did not exhibit a sharp descent, as it is a momentum oscillator rather than a trend indicator, showing the deviation of the total increase–decrease ratio within specific historical periods. In practical applications, MAD could be combined with RSI to enhance the accuracy of turning point detection, where MAD becomes positive and RSI goes over 50.

However, RSI failed to detect the turning point in h055. This result was because the trend line of h055 exhibited a continuous decline during the monitoring period, preventing it from intersecting with the SMA line, and RSI remained unchanged at 0. Extending the monitoring period, such as two weeks before the expected calving date, may enhance the effectiveness of our method for various pregnant individuals. MAD and MACD could still detect the turning point even in such cases, as they focus on detecting trend conversions. In financial trading, this phenomenon is called ‘divergence,’ where the price trend and the indicator’s direction are opposite. Divergence is known as a leading signal of a trend reversal and may further enhance the effectiveness of MAD and MACD in calving prediction.

Our study is an early example of applying financial technical indicators to analyze continuously updated scientific data. The prevalence of dynamic data has surged with the increasing use of IoT devices^11^, as such wireless vaginal temperature sensor^22^, which rapidly and automatically upload data to internet clouds. Given the wide array of financial technical indicators, numerous applicable indicators could be employed even in livestock-related studies. For instance, our calving date forecasting method could be adapted for other mammals such as pigs, horses, and sheep. Some technical indicators may also be useful in diagnosing various diseases when applied to monitoring data related to behavior, physiological phenomena, and genomic information^23^. While we used default settings (e.g., 12, 26 in MACD) for the three indicators in this study, adjusting these settings according to data characteristics may be necessary to detect the signals more effectively. In addition, we remark that machine learning methods may benefit from technical indicators.

Although our study focused on utilizing trends extracted from the original data through STL analysis to identify trend-turning points, it is worth noting that other aspects of the data, such as residuals and periodic fluctuations, could also provide valuable insights when analyzing different time-series data. Residuals, in particular, contain information about rapid changes over short periods and can be used to detect sudden shifts in monitoring targets^16^. For instance, rapid temperature fluctuations within a honeybee hive during a swarm event^24^ could be effectively detected using residuals. Periodic fluctuations may also be leveraged to identify changes in daily behavioral patterns^25,26^.

In conclusion, our study presents a simple and practical method for forecasting and alarming calving dates using STL analysis and financial technical indicators. The sufficient period of attaching the device should have been about two weeks before the calving date predicted by the breeding date. Considering the two troubled devices, non-contact heart rate monitoring using infrared and RGB video cameras may be another promising approach^27^. This study remains a feasibility study with limited observations, and further research should validate this method’s effectiveness with a larger dataset by providing a more accurate baseline HR. Additionally, exploring other technical indicators and various settings would provide valuable insights. While machine learning and AI technologies continue to advance rapidly, developing simple and practical solutions to the problems livestock farmers face may also be important.

## Methods

### Cattles

All procedures were approved by the Institute Committee for Animal Use and Care of the National Agriculture and Food Research Organization (NARO), Tsukuba, Japan, under protocol number 21C072ILGS, and performed in accordance with the relevant guidelines and regulations. The study was conducted at the National Institute of Livestock and Grassland Science, NARO facilities. This study is reported under ARRIVE guidelines for animal research.

We utilized eight pregnant Holstein cows, including three primiparous and five multiparous cows. The mean age of the cows was 58.9 ± 27.4 months, with the multiparous cows having a mean parity of 2.2 ± 1.2, calved between June and October 2021. Each cow individual was transported to a stanchion stall (1.6 × 1.2 m) approximately one and two months before their expected calving dates. None of these animals required veterinary assistance. The animals were fed twice a day at 09:00 and 16:00 with concentrate and chopped Italian ryegrass hay (*Lolium multiflorum*) to satisfy their nutrient requirements. Water and minerals were available ad libitum.

Electrocardiogram data of the cows were measured continuously, as described in a previous paper^28^. Electrocardiography (ECG) was performed using a Holter monitor (QR2500; Fukuda ME Co. Ltd, Tokyo, Japan) with an apex-base bipolar lead using five disposable skin-adhesive electrodes and conductive gel. Two sets of −/ + electrodes were attached on the upper part of the left scapula and the bottom of the left thorax, and a fifth electrode was placed in the middle as ground. A girth belt comprising a specialized pocket to accommodate the Holter monitor was engineered to protect the electrodes against external impact. The Holter monitor, electrodes, and belt were attached to the experimental cows seven days before their expected calving date. Two electrocardiograms at a sampling rate of 150 Hz were simultaneously recorded, and the one that generated less noise was used for subsequent analysis. The ECG power spectral analysis software (SRV-2W; Softron Co. Ltd, Tokyo, Japan) was used to resample the recorded ECG data at a sampling rate of 500 Hz. The software detected the R wave from the peak of the QRS complex of the ECG wave to calculate the HR and draw the R-R interval tachogram as raw HRV. Any R-R interval deviating from the average by more than 30% was excluded as an outlier.

The precise calving time (when the calf would be fully expelled) was obtained from video recordings (BA4M-J4DVR; CCTVJAPAN, Okayama, Japan). The R-R intervals were divided every 30 min and averaged for analysis. Data from two pregnant individuals were excluded from the analysis due to partial data loss in device troubles (h019 and h873). The missing values were imputed for these cases, and the imputed data were analyzed using the identical methods as written below. We show the results of the two cows in Supplementary Materials (Fig. S1, S2, Table S1).

### Data analysis

We decomposed the recorded data of R-R intervals from six individuals down into trend, seasonality, and remainder by STL analysis^16^. This STL analysis is based on the assumption that the trend in time-series data can be approximated by a LOESS continuous curve (span = 0.75, polynomial degree = 2, error model = "Gaussian"). In this study, the seasonality component represents a daily fluctuation in R-R intervals.

Subsequently, we employed financial technical indicators to identify turning points in the trends. We selected three widely used indicators: Moving Average Deviation Rate (MAD), Moving Average Convergence/Divergence (MACD), and Relative Strength Index (RSI)^18,19^. The calculation formulas for these indicators are outlined in Table 1. MAD and MACD are trend indicators known for their ability to assess trend direction, i.e., upward or downward. MAD calculates the deviation ratio between the current price and SMA, which is the average price of past prices, while MACD is the difference between short-term (default 12 periods, i.e., 6 h in this study) and long-term EMAs (default 26 periods, 13 h), with recent prices receiving more weight than older ones^18^. RSI is a momentum oscillator that gauges momentum (short trend) strength based on the ratio of upward to downward price changes over the past 14 days^19^. RSI values range from 0 to 100, with a value of 50 indicating equal total changes. Values above 50 suggest an upward momentum, while values below 50 indicate a downward momentum.

We set the number of periods for SMA at 12, corresponding to the average value over the past 6 h, considering that R-R trend values were recorded at 30-min intervals. Longer periods than 6 h could provide a clearer distinction between the trend and SMA values but would require at least that amount of time to generate the first SMA value. MAD represented the deviation ratio between the trend and SMA values. For MACD, we utilized default settings of 12 and 26 for short and long EMAs, respectively. MACD, with these settings, is good at detecting the trend for longer than 12 periods, not controlled by the short-term momentum of fewer than 12 periods. RSI was configured with a period of 14 (Table 1).

Using these indicators, we identified turning points in the R-R trend. We considered the peak of each indicator as the turning point, signifying a change in trend direction from upward to downward. We measured the remaining time from the signal at the peak generated by each indicator to calving. Data from cow h055 were excluded from the analysis because of no peaks by RSI. We utilized a generalized linear mixed model (GLMM) to compare the remaining time between the three indicators. In this model, indicator type served as the explanatory variable, remaining time as the response variable, and individual cow as a random effect. In cases where the effect of the indicator type was significant, we conducted multiple pairwise comparisons with Bonferroni correction (p < 0.05/3 = 0.0167). All statistical analyses were carried out using the open-source statistical software R version 4.1.2^29^ and the “lme4” package^30^.

## Supplementary Information

Supplementary Information.

## Data Availability

The data and codes supporting this study’s findings are available from the corresponding author upon reasonable request with the permission of NARO.
