# Supplementary material for: A feasibility study on predicting cow calving time over 40 h in advance using heart rate and financial technical indicators
Source: Sci Rep. 2024 Sep 18;14:21748. doi: 10.1038/s41598-024-72521-w (PMC11411094; doi:10.1038/s41598-024-72521-w)
Supplement: Supplementary file 1 — Supplementary Information. [file 41598_2024_72521_MOESM1_ESM.docx]

**Supplementary Materials**

We present the results of two individuals (h019, h873) removed from the main results because a part of the records was missing due to device troubles. In the total 240 records of the average R-R time-series data every 30 minutes in h019, the number of missing records was 22 (i.e., N.A.), while 13 in h873.

We first imputed the missing records to conduct the same analyses for the two datasets. By using the “imputeTS” package on R (Moritz & Bartz-Beielstein 2017), we fitted an autoregressive integrated moving average model with seasonality (Seasonal ARIMA) to each dataset because the results of STL analysis from the other six individuals suggested that those datasets folded the model assumptions.

Using the imputed datasets, we carried out STL analysis and calculated the three trading indicators (See details in the main text). Results are shown in the following figures and a table.

**Figure S1.**


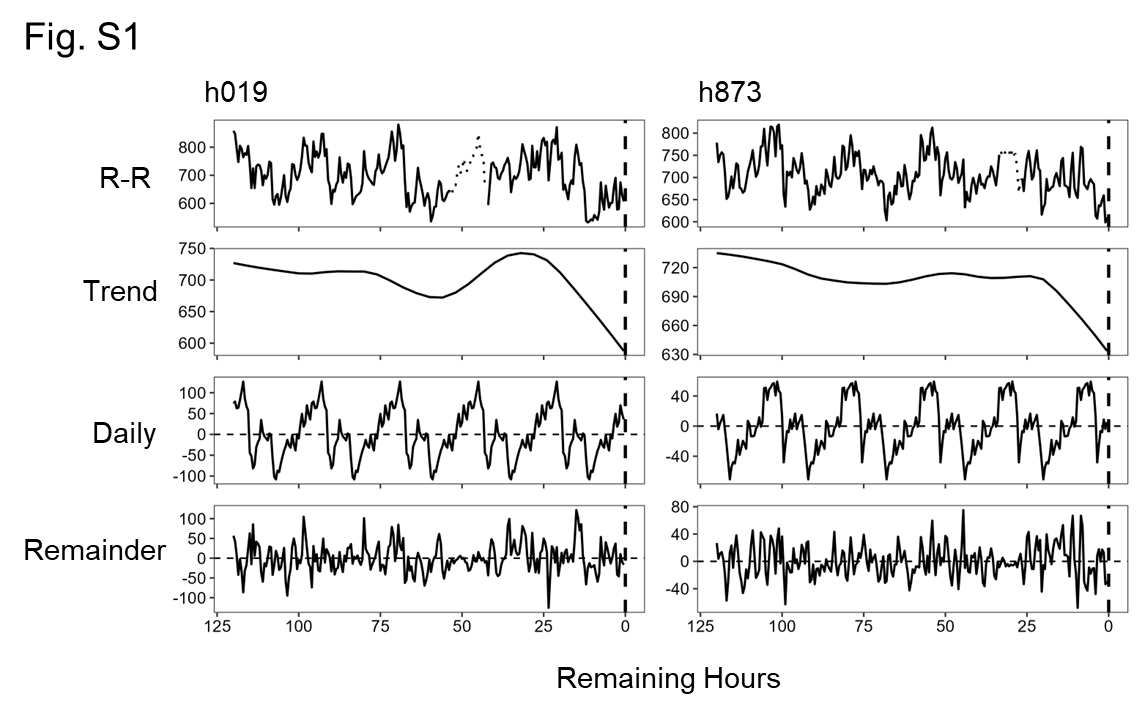


Figure S1. Time-series data of R-R intervals recorded till the calving of every individual (first row), and its trend (second), daily cycle (third), and the remainder (fourth) were decomposed by STL analysis. The dotted line in the original figures indicates the imputed values by the ARIMA model.

**Figure S2.**

Figure S2. The moving average deviation rate (MAD, the first row), moving average convergence/divergence (MACD, second row), and relative strength index (RSI, third row) of the R-R trend extracted by STL analysis in h019 and h873. A part of either data was missing due to device trouble and imputed by the ARIMA model. A horizontal dashed line was drawn at zero in MAD and MACD and at 50 in RSI.


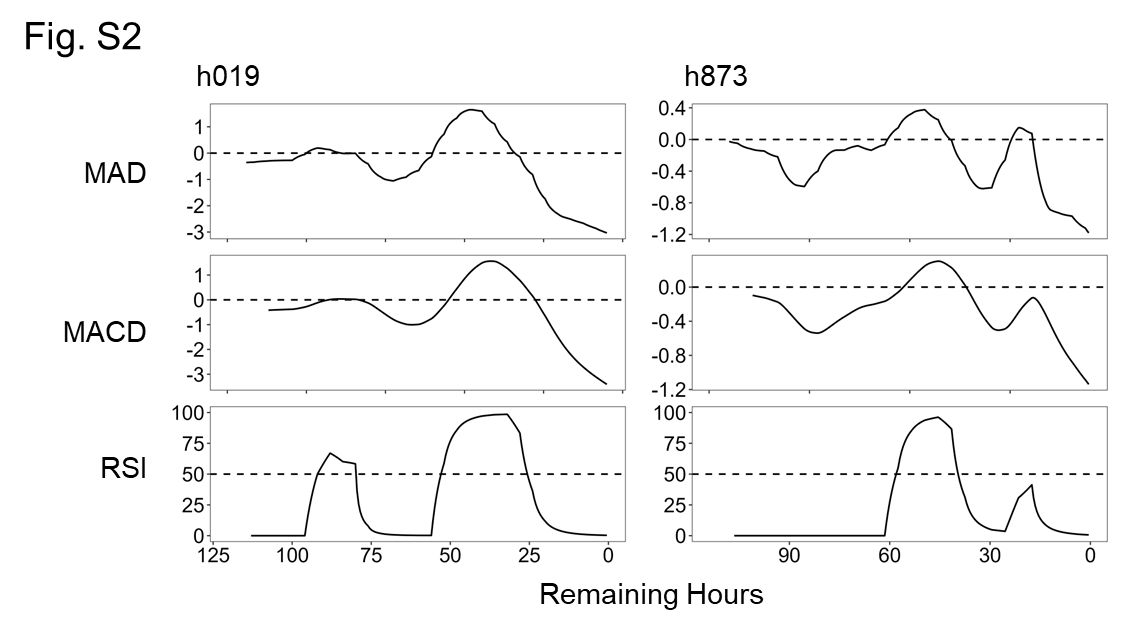


Table S1. Values of trading indicators and the remaining hours till calving at the turning point of the R-R trend.

|  | h019 | | h873 | |  |
| --- | --- | --- | --- | --- | --- |
| Indicator | Value | Hours | Value | Hours | Mean (S.D.) |
| SMA12 | 741.4 | 29.5 | 710.7 | 23.0 | 26.3 ± 4.6 |
| MAD12 | 10.8 | 39.5 | 0.7 | 28.0 | 33.8 ± 8.1 |
| MACD | 1.6 | 37.0 | 0.0 | 24.0 | 30.5 ± 9.2 |
| RSI | 98.6 | 32.0 | 73.5 | 24.0 | 28.0 ± 5.7 |
| Mean (S.D.) |  | 34.5 ± 4.6 |  | 24.8 ± 2.2 |  |

Even if some records were missing in the original, STL analysis decomposed the imputed data and showed similar results with the other six. The R-R trend rose and sharply descended in the datasets of h019 and h873 as well. Trading indicators highlighted the convex curve and signaled the descent. The average remaining time was 34.5 ± 4.6 (mean ± S.D.) and 24.8 ± 2.2 hours in h019 and h873, respectively. These times were shorter than the average time of the other six individuals, 56.1 ± 11.01 hours. However, there is no information on which imputation, the pregnant’s physical conditions, device troubles, or other factors caused the results.

**References**

Moritz S, Bartz-Beielstein T (2017). “imputeTS”: Time Series Missing Value Imputation in R. The R Journal, 9(1), 207–218. doi:10.32614/RJ-2017-009.
